# Muscle short-range stiffness behaves like a maxwell element, not a spring: Implications for joint stability

**DOI:** 10.1371/journal.pone.0307977

**Published:** 2024-08-14

**Authors:** Jeff M. Barrett, Masoud Malakoutian, Sidney Fels, Stephen H. M. Brown, Thomas R. Oxland

**Affiliations:** 1 Department of Orthopaedics, The University of British Columbia, British Columbia, Canada; 2 ICORD Research Centre, The University of British Columbia, British Columbia, Canada; 3 Department of Mechanical Engineering, The University of British Columbia, British Columbia, Canada; 4 Department of Electrical and Computer Engineering, The University of British Columbia, British Columbia, Canada; 5 Department of Human Health and Nutritional Sciences, University of Guelph, Guelph, Canada; Loughborough University, UNITED KINGDOM OF GREAT BRITAIN AND NORTHERN IRELAND

## Abstract

**Introduction:**

Muscles play a critical role in supporting joints during activities of daily living, owing, in part, to the phenomenon of short-range stiffness. Briefly, when an active muscle is lengthened, bound cross-bridges are stretched, yielding forces greater than what is predicted from the force length relationship. For this reason, short-range stiffness has been proposed as an attractive mechanism for providing joint stability. However, there has yet to be a forward dynamic simulation employing a cross-bridge model, that demonstrates this stabilizing role. Therefore, the purpose of this investigation was to test whether Huxley-type muscle elements, which exhibit short-range stiffness, can stabilize a joint while at constant activation.

**Methods:**

We analyzed the stability of an inverted pendulum (moment of inertia: 2.7 kg m^2^) supported by Huxley-type muscle models that reproduce the short-range stiffness phenomenon. We calculated the muscle forces that would provide sufficient short-range stiffness to stabilize the system based in minimizing the potential energy. Simulations consisted of a 50 ms long, 5 Nm square-wave perturbation, with numerical simulations carried out in ArtiSynth.

**Results:**

Despite the initial analysis predicting shared activity of antagonist and agonist muscles to maintain stable equilibrium, the inverted pendulum model was not stable, and did not maintain an upright posture even with fully activated muscles.

**Discussion & conclusion:**

Our simulations suggested that short-range stiffness cannot be solely responsible for joint stability, even for modest perturbations. We argue that short-range stiffness cannot achieve stability because its dynamics do not behave like a typical spring. Instead, an alternative conceptual model for short-range stiffness is that of a Maxwell element (spring and damper in series), which can be obtained as a first-order approximation to the Huxley model. We postulate that the damping that results from short-range stiffness slows down the mechanical response and allows the central nervous system time to react and stabilize the joint. We speculate that other mechanisms, like reflexes or residual force enhancement/depression, may also play a role in joint stability. Joint stability is due to a combination of factors, and further research is needed to fully understand this complex system.

## Introduction

Mechanical stability refers to the property of a system to resist disturbances and return to a state of equilibrium. Many joints in the human body are prone to instability; for example, the human spine is inherently unstable and buckles even under modest loads. Specifically, the lumbar spine without its muscles has a compressive critical load of 88 N, while the cervical spine buckles around 11 N under similar conditions [1–3]. The fact that these loads are on the order of magnitude of the head’s weight (~60 N) alone highlights the substantial role of the muscles, which provide the forces required to hold static postures and the stiffness to ensure their stability [4–11].

**Fig 1:**
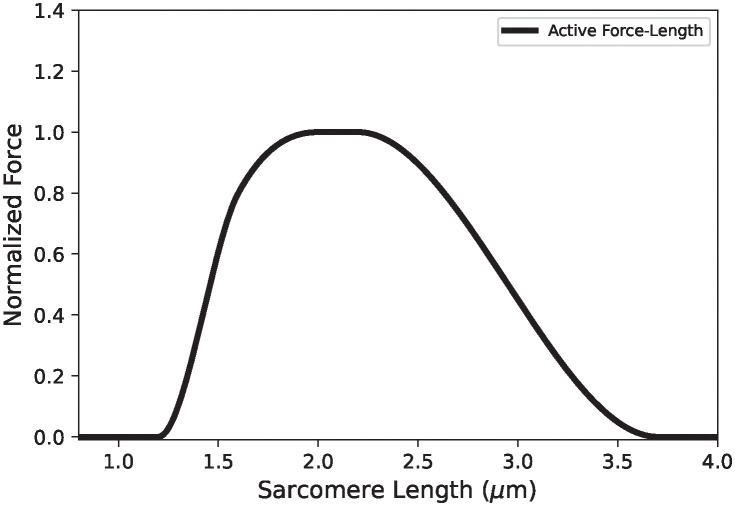
The active force-Length curve demonstrating the difference between transient and static stiffness. If a muscle at point A is rapidly lengthened or shortened, then the force produced follows the curve labelled *k* + *k*′ (i.e. with stiffness *k* + *k*′) rather than *k* (i.e. with stiffness *k*).

Muscle stiffness has long been considered a significant contributor to stability, often represented as static springs [6,10,12–16]. In this manuscript it is worth distinguishing active static from transient (or short-range) muscle stiffness. Gordon, Huxley & Julian (1966) [17] first described the canonical force-length relationship that relates a muscle’s steady-state force to its length following an isometric contraction (Fig 1). The curve shows an ascending region at low lengths, a plateau region, and a linear descending limb at longer lengths. The apparent negative stiffness of the descending limb suggests that muscles in this region may be prone to instability [18]. However, during a perturbation, the muscles are not in steady state. Instead, the actin-bound myosin cross-bridges stretch before release as the active muscle lengthens. This mechanism, called ‘short-range stiffness,’ produces a transient force larger than the steady-state force-length curve during lengthening (Fig 1) [19]. Thus, even if the muscle’s steady-state force decreases, its transient stiffness is still positive [20].

Short-range stiffness provides an attractive mechanical explanation for ensuring joint stability since it occurs immediately and without input from the central nervous system [5,9,21]. These arguments follow a similar line of reasoning that Allinger et al. [20] proposed to stabilize sarcomeres in series during lengthening contractions on the descending limb of the force-length relationship. However, Zahalak [22] used a simplified viscoelastic model to show that short-range stiffness alone cannot resolve serial sarcomere instability. To date, there has not been a similar analysis to demonstrate how short-range stiffness may contribute to joint-level stability.

There are two dominant models of force production in muscle: Hill-type and Huxley-type. The Hill-type approach is a phenomenological model that, due to its simplicity, dominates most macroscopic investigations [23–27]; however, contractile element short-range stiffness is often omitted from this modelling approach. Previous investigations with Hill-type models typically treat the muscles as static springs [6,28,29], or use a modified Hill-type framework with various arrangements of springs and dashpots to achieve the short-range stiffness effect [16,30]. By stark contrast, the Huxley model is more mechanistic in nature, and begins by considering the reaction dynamics of a population of actin-myosin proteins in a half-sarcomere [31,32]. Many other muscle properties can be derived from this mechanistic description, for instance, the force-velocity relationship, heat-of-shortening, and, most pertinent to this analysis, short-range stiffness [33].

We use the Lyapunov notion of stability applied to equilibrium points of dynamical systems throughout this manuscript, which classifies them as *stable* or *asymptotically stable* [10,34]. An equilibrium is considered stable if for any specified fixed distance to the equilibrium, there exists a neighbourhood of initial conditions whose trajectories will stay within that fixed distance. Asymptotic stability is a stronger condition, and requires that the equilibrium not only be stable, but that all trajectories that begin nearby the equilibrium point converge to that equilibrium. A simple harmonic oscillator is an example of a system that is stable, but not asymptotically stable, as it will oscillate around its equilibrium point and never converge. On the other hand, a damped harmonic oscillator is asymptotically stable, as the damped oscillations decrease exponentially toward equilibrium. For a mechanical system with *n*-degrees of freedom, if all the forces acting on it are conservative, then its stable, but not asymptotically stable, equilibria occur at minimums in the potential energy function. This follows because near an equilibrium point the system behaves like *n*-decoupled simple harmonic oscillators [35], and we refer to this special case as *classical stability*.

In this article, we consider the stability of an inverted pendulum supported by Huxley-type muscle model elements that portray the short-range stiffness phenomenon. We hypothesized, based on numerous previous classical stability analyses [4,6], that these muscles could maintain stability about an upright posture provided they sufficiently co-contracted.

## Methods

**Fig 2:**
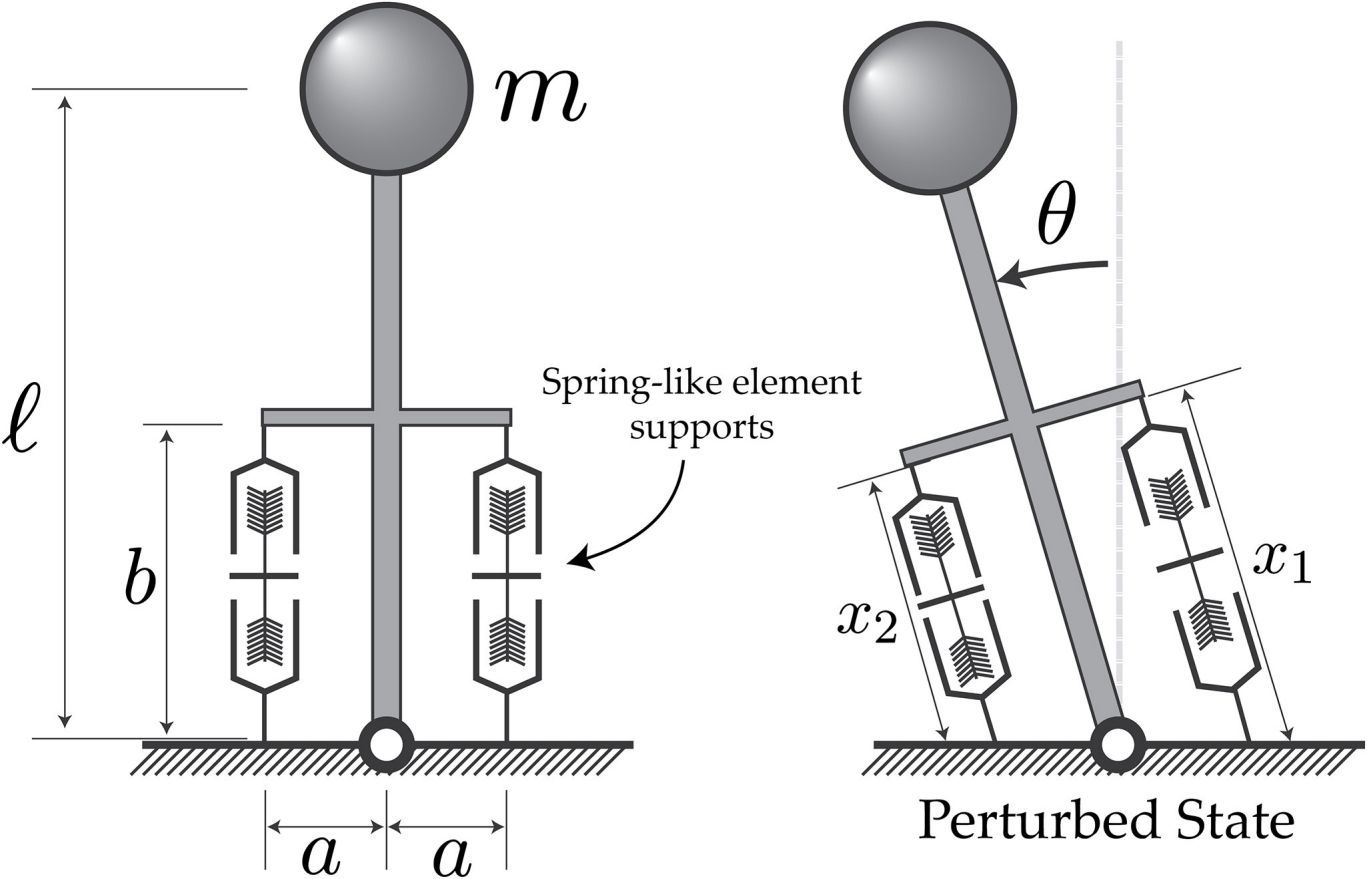
Simplified inverted pendulum model for this analysis. The pendulum has a length *ℓ*, mass *m*, and is supported by two spring-like elements of length *b* on either side of the pin-joint. They have moment arms ±*a* and are aligned with the pendulum’s long axis in the upright position. On the right is the pendulum during the perturbation, with its angle relative to vertical indicated by *θ*, and muscle lengths by *x*_1_ and *x*_2_.

The system under consideration is a single degree-of-freedom inverted pendulum supported, on its lateral sides, by springlike elements (Fig 2) to represent the musculature [4–6]. Under this paradigm, the force in the springs is taken to be the muscles’ forces. The challenge is to find a set of muscle forces, stiffnesses, or activations, that stabilize an upright position. We first derive some general conditions on the force and stiffness of the muscles required for stability, before exploring potential muscle models that can be used to simulate this situation.

### Preliminary calculations

Under classical stability analysis, an equilibrium is stable if and only if the potential energy is at a minimum. To analyze this system, we suppose that each of the muscles has a length-dependent potential energy function. Defining *x*_1_ and *x*_2_ as the lengths of muscle 1 and muscle 2, respectively, and letting the pendulum mass be *m* with length *ℓ*. Under these assumptions, we have the potential energy function:

$$V\left(\theta \right)=V_{1}\left(x_{1}\right)+{\text{V}}_{2}\left(x_{2}\right)+mgl\;\text{cos}\;\theta$$ (1)

Where *m* and *ℓ* are the mass and length of the pendulum, respectively, and *g* is the acceleration due to gravity (9.81 m/s^2^). The spring potential energies, *V*_1_ and *V*_2_, are functions of the spring lengths, *x*_1_ and *x*_2_, which are themselves functions of the pendulum’s vertical angle, *θ*. For convenience, we suppose that the origins of the muscles are at *O*_*i*_ = (±*a*, 0), and that the insertions are both *b* units vertical from their origins onto the inverted pendulum in the neutral configuration. This gives closed-form expressions for their lengths:

$$x_{1}\left(\theta \right)=\sqrt{b^{2}+2a^{2}\left(1-\text{cos}\;\theta \right)+2ab\;\text{sin}\;\theta }$$ (2a)

$$x_{2}\left(\theta \right)=\sqrt{b^{2}+2a^{2}\left(1-\text{cos}\;\theta \right)-2ab\;\text{sin}\;\theta }$$ (2b)

An important property derivable from these lengths are the springs’ moment arms, which are the derivatives of these functions with respect to the angle, *θ*:

$$r_{1}\left(\theta \right)=\frac{\partial x_{1}}{\partial \theta }=\frac{a^{2}\;\text{sin}\;\theta +ab\;\text{cos}\;\theta }{x_{1}},\;r_{2}\left(\theta \right)=\frac{\partial x_{2}}{\partial \theta }=\frac{a^{2}\;\text{sin}\;\theta -ab\;\text{cos}\;\theta }{x_{2}}$$ (3)

Since we are analyzing the system about *θ* = 0, it is also helpful to define the length (*L*) and moment arms (*r*) of the springs in that position:

$$L=x_{1}\left(0\right)=x_{2}\left(0\right)=b,\;r=r_{1}\left(0\right)=-r_{2}\left(0\right)=a$$ (4)

A final useful observation we can make about this system is that, in an upright position, the second derivative of the spring lengths vanishes.


$${\left.\frac{\partial^{2}x_{1}}{\partial \theta^{2}}\right|}_{\theta =0}={\left.\frac{\partial^{2}x_{2}}{\partial \theta^{2}}\right|}_{\theta =0}=0$$ (5)

### Classical stability analysis

For this system to be stable in an upright position (*θ* = 0), requires that the gradient of Eq 1 vanishes, and that its second derivative is positive. Using the chain-rule, we can evaluate the first derivative:

$$\frac{dV}{d\theta }=V_{1}^{\prime }\left(x_{1}\right)r_{1}+V_{2}^{\prime }\left(x_{2}\right)r_{2}-mgl\;\text{sin}\;\theta$$ (6)

Where we have used Eq 3 to rewrite the derivatives of *x*_*i*_ with respect to angle as the moment arms. We require that the upright position is in equilibrium, so that Eq 6 evaluated at *θ* = 0 vanishes. Eq 6 can be further simplified by recognizing that the muscles’ force, *F*_*i*_, is the gradient of the potential energy, or that $F_{i}=V_{i}^{\prime }\left(x_{i}\right)$. Doing so results in the familiar moment-balance required for equilibrium:

$${\left.\frac{dV}{d\theta }\right|}_{\theta =0}=0=-aF_{1}+aF_{2}$$ (7)

We next consider the second derivative of Eq 1, which needs to be positive to ensure stability. We evaluate the derivative of Eq 6 using the product and chain rules:

$$\frac{d^{2}V}{d\theta^{2}}=V_{1}^{\prime \prime }\left(x_{1}\right)r_{1}^{2}+V_{1}^{\prime }\left(x_{1}\right)\frac{\partial r_{1}}{\partial \theta }+V_{2}^{\prime \prime }\left(x_{2}\right)r_{2}^{2}+V_{2}^{\prime }\left(x_{2}\right)\frac{\partial r_{2}}{\partial \theta }-mgl\;\text{cos}\;\theta$$ (8)

Because of the geometry of the pendulum, notably Eq 5, the terms involving the derivatives of the moment arms with respect to angle vanish once we evaluate this expression at *θ* = 0. Further, we recognize the muscles’ stiffness, $K_{i}=\frac{dF_{i}}{dx_{i}}=V_{i}^{\prime \prime }\left(x_{i}\right)$, which yields the stability condition:

$${\left.\frac{d^{2}V}{d\theta^{2}}\right|}_{\theta =0}=a^{2}K_{1}+a^{2}K_{2}-mgl>0$$ (9)

Together, Eqs 7 and 9 place restrictions on the muscle forces and stiffnesses required to maintain stability in the upright position.

### Force-stiffness relationships and the Hill model

So far, this analysis has been very general with no assumptions made about the nature of the springs supporting the inverted pendulum. For skeletal muscles, Bergmark (1989) [6] used a relationship between the muscles’ active force (*F*), length (*x*), and stiffness (*K*) derived from cross-bridge theory, which we call the force-stiffness relationship [30,33,36]:

$$K=q\frac{F}{x}$$ (10)

Where *q* is a dimensionless constant of proportionality, often chosen between 1.0 and 40 [6,14,15,37–39]. Using this relationship, Eqs 7 and 9 can be recast as constraints on muscle forces [40,41]. For our simple pendulum model, equilibrium (Eq 7) requires that both spring forces are equal, and stability (Eq 9) requires that:

$$F>\frac{mglb}{2qa^{2}}$$ (11)

This muscle force solution was a triumph of this stability approach since Eq 11 predicts considerable antagonistic muscle co-contraction to stabilize an upright posture in the absence of a net joint moment. Supporting this prediction are countless *in vitro* and *in silico* studies which have demonstrated that the spine is inherently unstable when it is not supported by active musculature [2,5,8,41–43].

However, the relationship in Eq 11 is problematic for forward-dynamics modelling purposes because the basic Hill-type muscle model simply does not capture short-range stiffness [44]. Consider a Hill-type muscle model, omitting the passive structures for brevity. The muscle’s force is given by the product of its activation (*α*), together with the maximum isometric force (*F*_0_), the force-length (*f*_*L*_) and force-velocity (*f*_*V*_) relationships [24,45]:

$$F_{i}=\alpha \;F_{0}\;f_{L}\left(x_{i}\right)\;f_{V}\left({\dot{x}}_{i}\right)$$ (12)

One can show that, since stiffness is the derivative of this expression with respect to *x*_*i*_, the Hill model is only consistent with the force-stiffness relationship if its force-length relationship is a power law (*i*.*e*. *f*_*L*_(*x*) = *cx*^*q*^, where *c* is a constant of integration and *q* is the dimensionless constant earlier). Unfortunately, this result may only be locally consistent with experimental evidence, and thus precludes a conventional Hill-type muscle model from exhibiting the short-range stiffness phenomenon that theoretically stabilizes the upright pendulum. Resolving this issue requires a departure from conventional Hill-type models, and exploring the mechanisms that give rise to Eq 10 in the first place.

### The Huxley model

An alternative to the Hill-type muscle model is the Huxley muscle model that is the foundation of the sliding filament theory [31]. Although it is more widely used to investigate muscle function at the molecular level [46–48], it has been used in several investigations at the whole muscle level [32,49–52]. We briefly describe the expression for the Huxley model with two-states, analyze some important results, and demonstrate that the Huxley model satisfies the force-stiffness relation.

**Fig 3:**
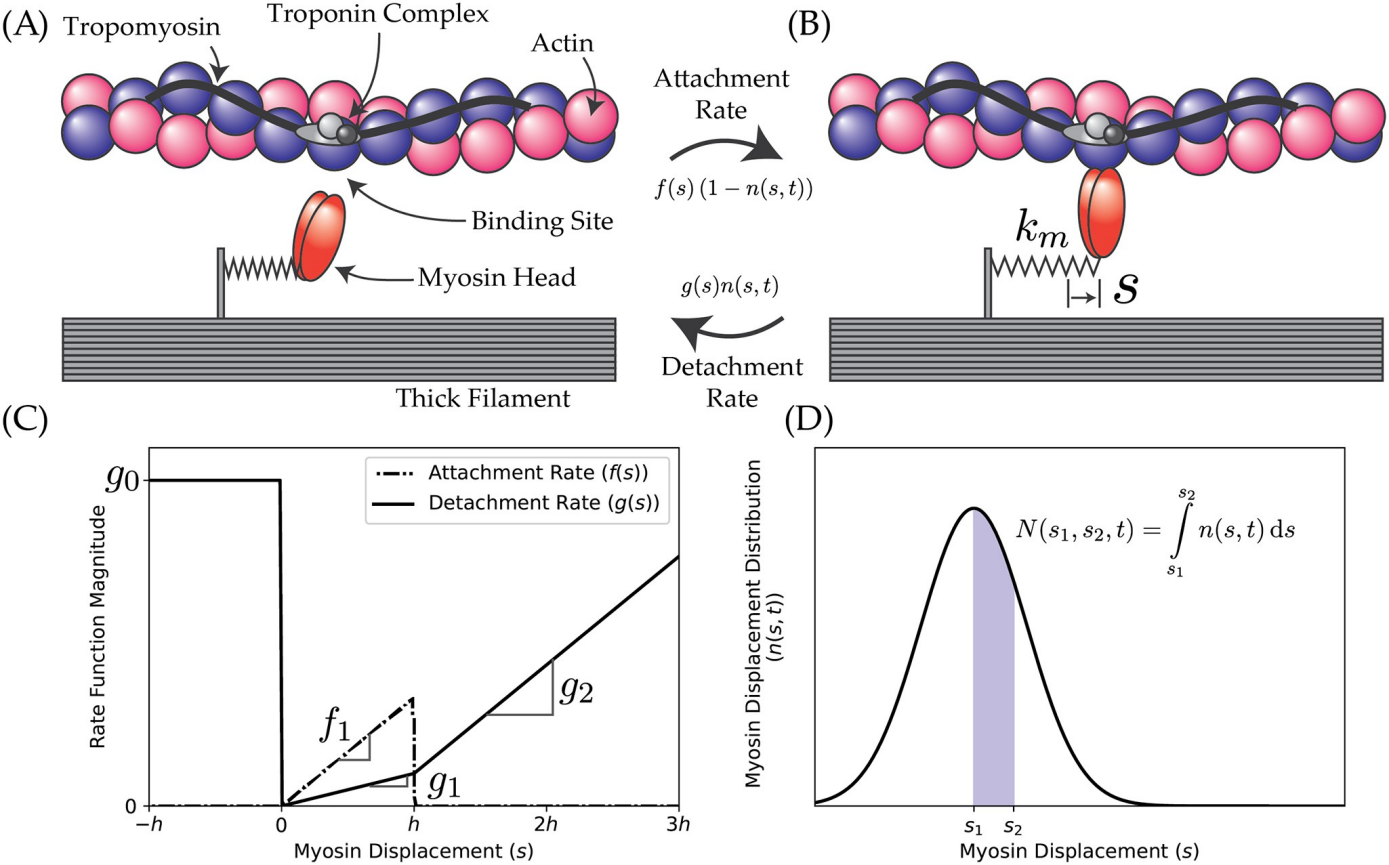
An overview of the two-state Huxley model used in this investigation. The myosin heads can exist in two states: (A) unbound, or (B) bound, to actin. Once bound, the model tracks the displacements among the myosin molecules, which have an associated stiffness of *k*_*m*_ ≈ 0.2 to 5.0 pN/nm. The rates between these two states are characterized by an attachment rate, parameterized by *f*(*s*), and a detachment rate, parameterized by *g*(*s*), both of which are graphically depicted in (C). A hypothetical displacement distribution function (D), where the area under this graph between displacements *s*_1_ and *s*_2_ is approximately the proportion of myosin heads that are bound with displacements between *s*_1_ and *s*_2_.

The Huxley model describes a half-sarcomere, where a large population of myosin-actin cross-bridges reside (Fig 3A). Each bound cross-bridge has its own displacement from an equilibrium position, *s* (Fig 3B), and can alternate between bound and unbound states through the attachment and detachment rate functions, *f*(*s*) and *g*(*s*), respectively (Fig 3C). The model considers only bound cross-bridges, which is used to construct a displacement distribution function over all the cross-bridges, *n*(*s*, *t*) (Fig 3D). This is so that *n*(*s*, *t*)Δ*s* can be thought of as, roughly, the number of cross-bridges that have stretches between *s* and *s* + Δ*s*. The time-evolution of *n*(*s*, *t*) is given by the partial differential equation [52]:

$$\frac{\partial n}{\partial t}-v\frac{\partial n}{\partial s}=\alpha f\left(s\right)\;\left(f_{L}\left(x\right)-n\left(s,t\right)\right)-g\left(s\right)\;n\left(s,t\right)$$ (13)

Where *α* and *f*_*L*_(*x*) are, as before, the muscle’s activation and force-length curve, and *v* is the half-sarcomere’s shortening velocity. Myosin cross-bridges possess a ‘characteristic stretch,’ denoted *h*, typically on the order of 5–10 nm [53], beyond which the reaction kinetics favour the detached state (Fig 3C).

### Force and stiffness in the Huxley model

Zahalak (1981) [49] presented expressions that map between the bond distribution, *n*(*s*, *t*) and macroscopic variables like the muscle’s force and stiffness. Notably, in our Supplemental Material, we follow a modified approach to Blangé et al., (1972) [33], the Huxley model satisfies the force-stiffness relationship, and is thus a candidate for stabilizing the inverted pendulum. At any instant, the muscle’s active force (*P*) is given by the expression:

$$P=\Gamma \overset{\infty }{\underset{-\infty }{\int }}s\;n\left(s,t\right)\;\text{d}s$$ (14)

Where Γ is a constant that depends on the sarcomere length, myosin stiffness—generally on the order of 0.2 to 5 pN/nm [19,54,55]—myosin concentration, cross-sectional area, and spacing between actin-myosin binding sites [49]. The integral in Eq 14 can be thought of as adding up the contributions to the total force from all the currently bound cross-bridges with Γ playing the role of an effective cross-bridge stiffness. Similarly, the stiffness (*K*) of the half-sarcomere is given by another integral [32]:

$$K=\frac{\Delta P}{\Delta x}=\frac{\Gamma }{2N_{S}}\overset{\infty }{\underset{-\infty }{\int }}n\left(s,t\right)\;\text{d}s$$ (15)

Where *N*_*s*_ is the number of sarcomeres arranged in series along the muscle fibre. An important solution for the Huxley model is the steady state ($\frac{\partial n}{\partial t}=0$) isometric (*v* = 0) condition, which is a uniform distribution. One can plug this analytic solution into Eqs 14 and 15 to derive the force-stiffness equation (see Supplemental Material):

$$K_{iso}=\frac{s_{0}}{h}\frac{P_{iso}}{x_{0}}=q\frac{P_{iso}}{x_{0}}$$ (16)

Which is similar to Eq 10, and identical in the case when the pendulum is upright. This property of the Huxley model gives rise to the short-range stiffness phenomenon. Of note, the theoretical dimensionless *q* is the ratio of the sarcomere length (~2.8 μm) [56] to the characteristic bond length (~5–10 nm) [57], which yields values of approximately 240–560 [16]: more than tenfold larger than previous investigations have assumed [6,30,36,58]. In generalizing this expression for other activations, we find that the short-range stiffness is tunable based on muscle activation, which is consistent with other investigations [44,59].

### Simulations

Numerical simulations were carried out in ArtiSynth [60], where custom muscle elements were programmed that incorporated a Distribution Moment (DM) approximation to the Huxley model. In particular, we used Donovan’s uniform distribution approximation [61], as it has the advantage of having the same steady-state behaviour as the underlying Huxley model.

The inverted pendulum had a mass of 30.0 kg and a length of 0.30 m, supported, by two 0.35 m long muscles (fibre lengths of 14 mm) with 0.05 m long moment arms, on either side of the single degree-of-freedom pivot joint (Table 1). With the muscle parameters in Table 1, along with the classical stability analysis (Eqs 7, 9 and 11), gives that the pendulum is stable if the muscle activations exceed 0.7%. To test this threshold, we ran simulations where the muscle activations were 0.5%, 1.0%, 10%, 50% and 100%. The pendulum was initially upright and, after one second, a 5 Nm torque was applied to the pendulum for 50 ms to test the stability of the pendulum in the numerical simulation. In addition, we compared simulation results to an unsupported inverted pendulum and one with equivalent linear springs with stiffnesses that match the muscle at 100% activation to give an example of a stable solution, and another where with the same springs and an additional damper in parallel with damping coefficient arbitrarily set to 10 Ns/mm, to give context on an asymptotically stable solution.

**Table 1:** Parameters that were used throughout the simulations. The muscle properties were selected to be representative of average muscle properties for lumbar spine extensors (erector spinae).

Parameter	Magnitude	Description	Reference
*m*	30 kg	Pendulum mass (~50^th^ percentile)	[62]
*ℓ*	0.3 m	Pendulum length (~50^th^ percentile)	[62]
*a*	0.05 m	Muscle moment arms	[63]
*b*	0.35 m	Muscle length	[64]
*L*_0_	0.14 m	Muscle Fibre Length	[64]
*f*_1_	15.0 s^-1^	Binding-rate slope	[57]
*g*_0_	170.0 s^-1^	Unbinding rate in compression	[57]
*g*_1_	8.0 s^-1^	Unbinding rate at low extensions	[57]
*g*_2_	25.0 s^-1^	Unbinding rate at high extensions	[57]
*s*_0_	2.2 *μ*m	Sarcomere length in upright posture	[57]
*h*	5 nm	Characteristic bond length	[57]
*ℓ*_*act*_	36 nm	Distance between actin binding sites	[57]
*k*_*m*_	5.0 pN/nm	Cross-bridge stiffness	(Campbell et al., 2018; Ford et al., 1981)
*M*_*m*_	460 *μ*M	Myosin concentration*	NA
*A*_0_	32 cm^2^	2x Physiological Cross-Sectional Area (Erector Spinae)	[64]

* Estimated so that the Huxley model would have a specific tension of 35 N/cm^2^, which is often selected for macroscopic biomechanical models [23].

## Results

**Fig 4:**
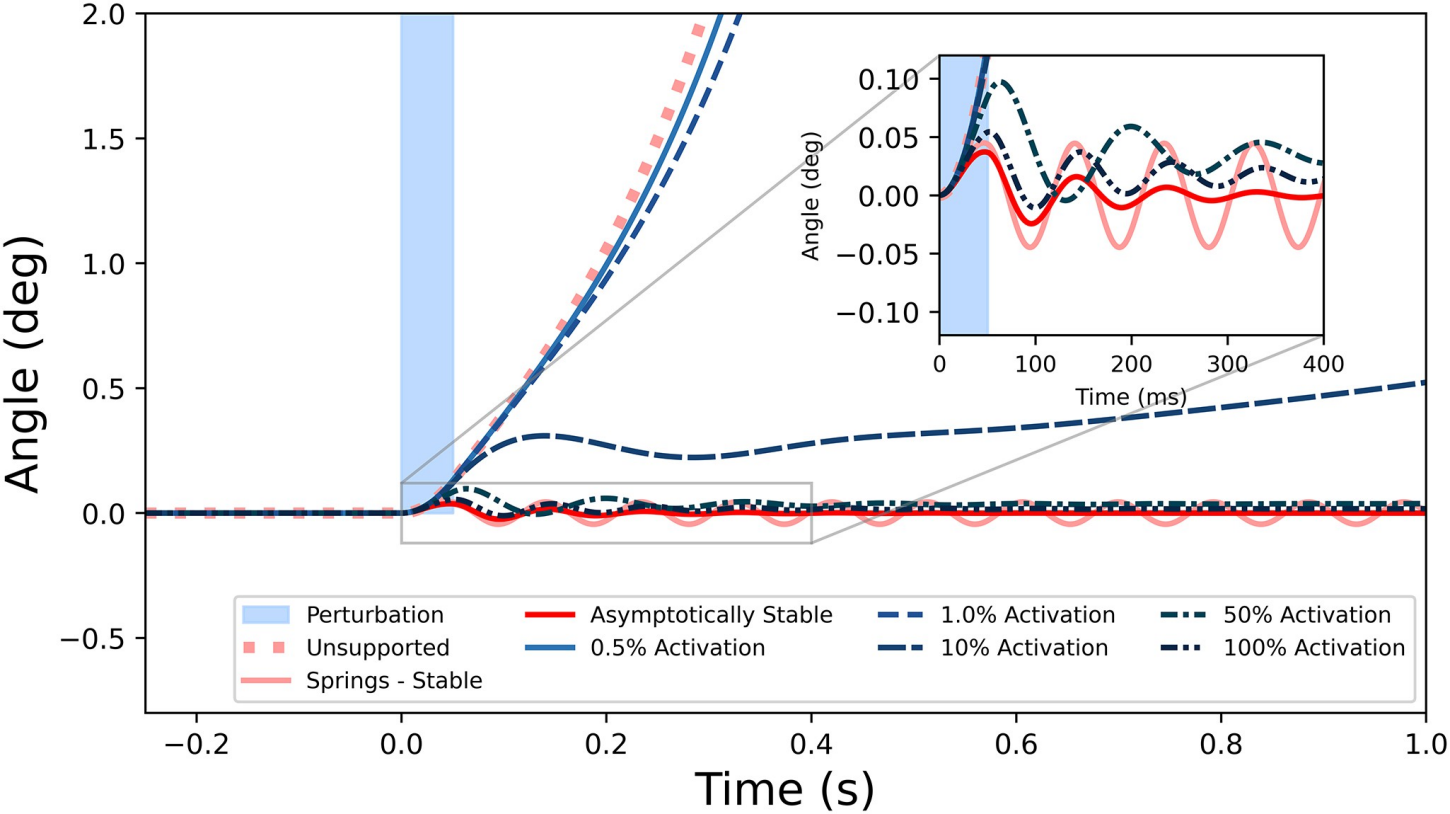
Angle time-histories before, during, and after the 50 ms, 5 Nm perturbation (shaded region). Constant muscle activation, even with short-range stiffness, was unable to stabilize the pendulum as evidenced by its eventual loss of equilibrium. The dashed linear spring represents muscles as springs whose stiffness matched the 100% activation short-range stiffness. For comparison is a damped oscillation which is considered asymptotically stable; one second after the perturbation even with high activations, the Huxley models did not return to the upright configuration.

**Fig 5:**
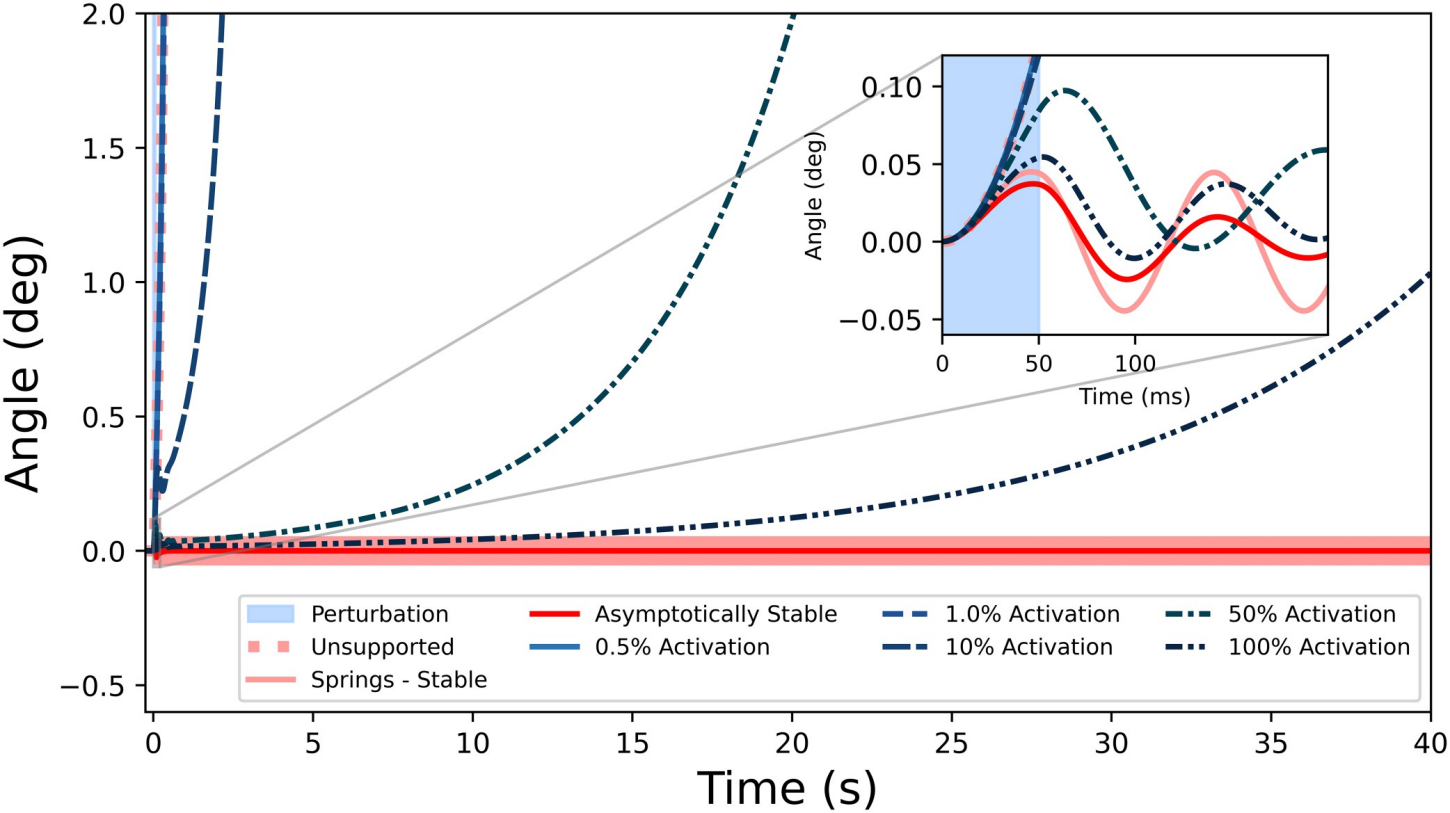
Longer-time simulations showing that the high activations (50% and 100%) eventually fell over in finite time. For comparison there is the stable case from the springs, and an asymptotically stable case from springs with a dashpot included for comparison.

During the timespan of the perturbation (0–50 ms), the corresponding angle-over-time curves were indistinguishable from the unsupported simulation. Supporting the pendulum with linear springs resulted in the expected oscillatory motion near the upright equilibrium position. However, none of the muscle activations managed to permanently stabilize the inverted pendulum, in the sense that the pendulum does not return to its equilibrium configuration (Fig 4). Even with maximally activated muscles, the pendulum eventually fell following the perturbations (Fig 5), over the course of tens of seconds. Animations of these simulations are included as Supplementary Material.

**Fig 6:**
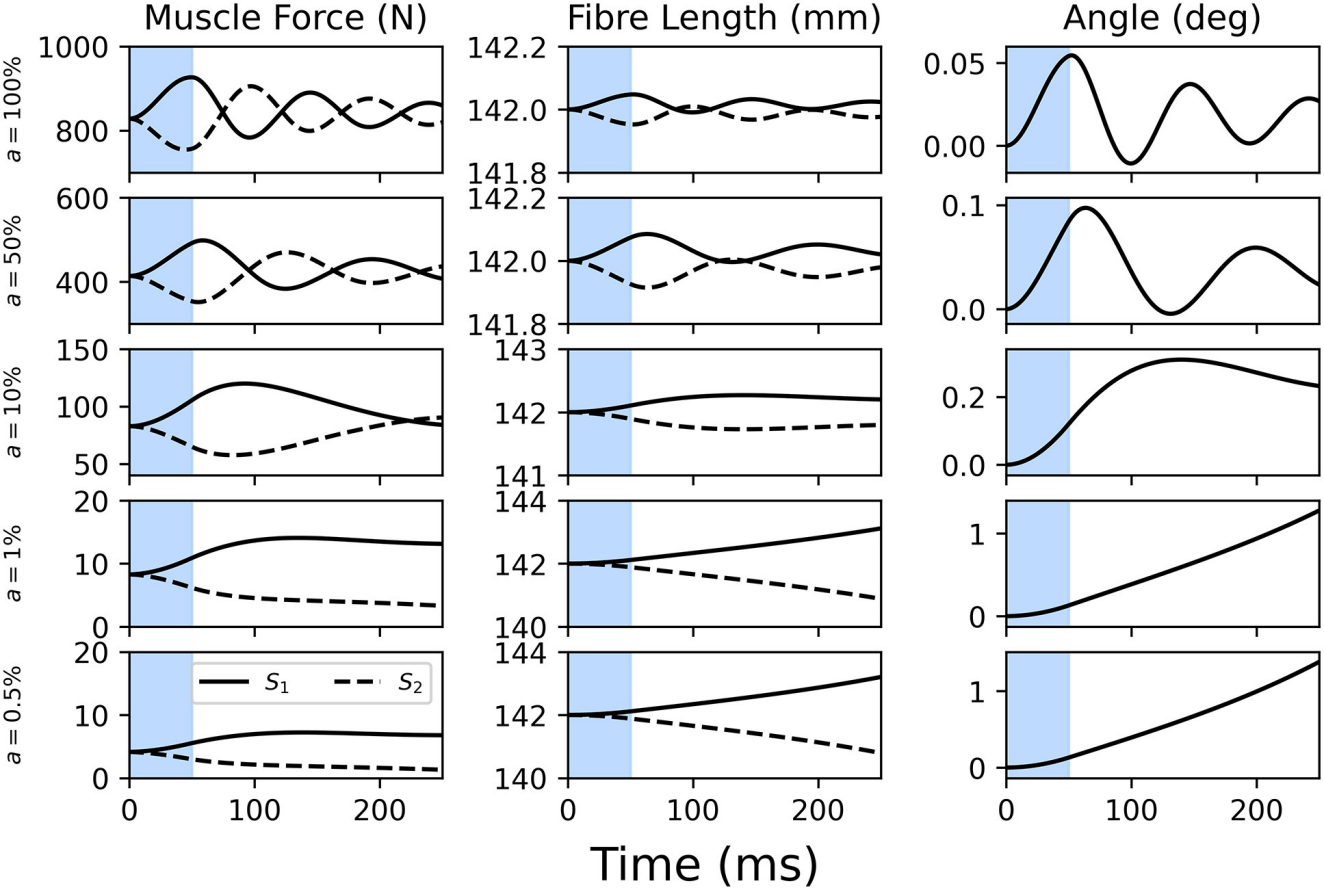
Muscle force (left column), fibre lengths (middle column) and pendulum angles (right column) for perturbations of varying muscle activations (rows) 250 ms after the initiation of the perturbation. At 50% or 100% activation, the pendulum oscillates, and the muscles alternate between concentric and eccentric loading. When each muscle is lengthening, its force is amplified, and diminished when shortening.

Muscle forces responded analogously to the pendulum angles, with higher activations predicting oscillations between concentric and eccentric contractions, until a quasi-steady state was reached (Fig 6). During low activations (<10%), the pendulum was moved away from its equilibrium within 250 ms.

## Discussion

In this investigation, we constructed an inverted pendulum model supported by muscle elements that portray the short-range stiffness phenomenon. Several investigators have argued that this muscle property contributes to joint stability (Cholewicki and McGill, 1996 [4]; Stokes and Gardner-Morse, 1995 [9]), and explains the antagonistic co-contraction observed in occupational and athletic tasks. However, our simulations suggest that the short-range stiffness phenomenon alone cannot, in isolation, ensure joint mechanical stability. Supporting this, our simulations with the largest magnitude of co-contraction with a modest perturbation force resulted in the eventual loss of equilibrium in the inverted pendulum model. In this section we explain why the Huxley model failed to ensure mechanical stability. We then offer a more nuanced conceptual model of short-range stiffness and suggest other physiological and neuromechanical mechanisms that may contribute to joint stability.

When an active muscle lengthens, the bound cross-bridges in its sarcomeres are stretched, causing them to sustain greater levels of force [19]. This is the physiological mechanism that underpins short-range stiffness, and it has led to the conceptual model that active muscles behave like mechanical springs. Unfortunately, if this were the whole story, then our inverted pendulum model could theoretically be supported by Huxley muscles that exhibit short-range stiffness. Indeed, using the stable equilibrium conditions (Eqs 7 and 9), it is relatively easy to design linear spring parameters—rest lengths and stiffnesses—that ensure the stability of our inverted pendulum. Since this behaviour is qualitatively different from our muscle model, we propose a revised conceptual model for short-range stiffness, more nuanced than purely spring-like behaviour. Our reasoning is that the extra displacement sustained by bound cross-bridges also increases their rate of detachment from actin, meaning that the added force they sustain is only transient. Rather than spring-like behaviour, the short-range stiffness phenomenon behaves more like a Maxwell element: a spring with a viscous dashpot arranged in series [22,65,66]. This result can be derived from Huxley’s Equation, which is included as Supplemental Material. Interestingly, this Maxwell element approximation has previously been used in the literature, although generally given phenomenological justifications and rarely derived from a cross-bridge model [18,22,47,67]. Further, including the muscle force-length relationship, Huxley’s model can be approximated as a standard linear viscoelastic solid, where the spring in parallel with the Maxwell element has force-elongation properties akin to the force-length curve. At constant activation, when this model is stretched slowly, it follows the force-length relationship; when it is stretched quickly, the dashpot does not have time to deform and so there is a transient increase in force.

### A related analytical stability analysis

**Fig 7:**
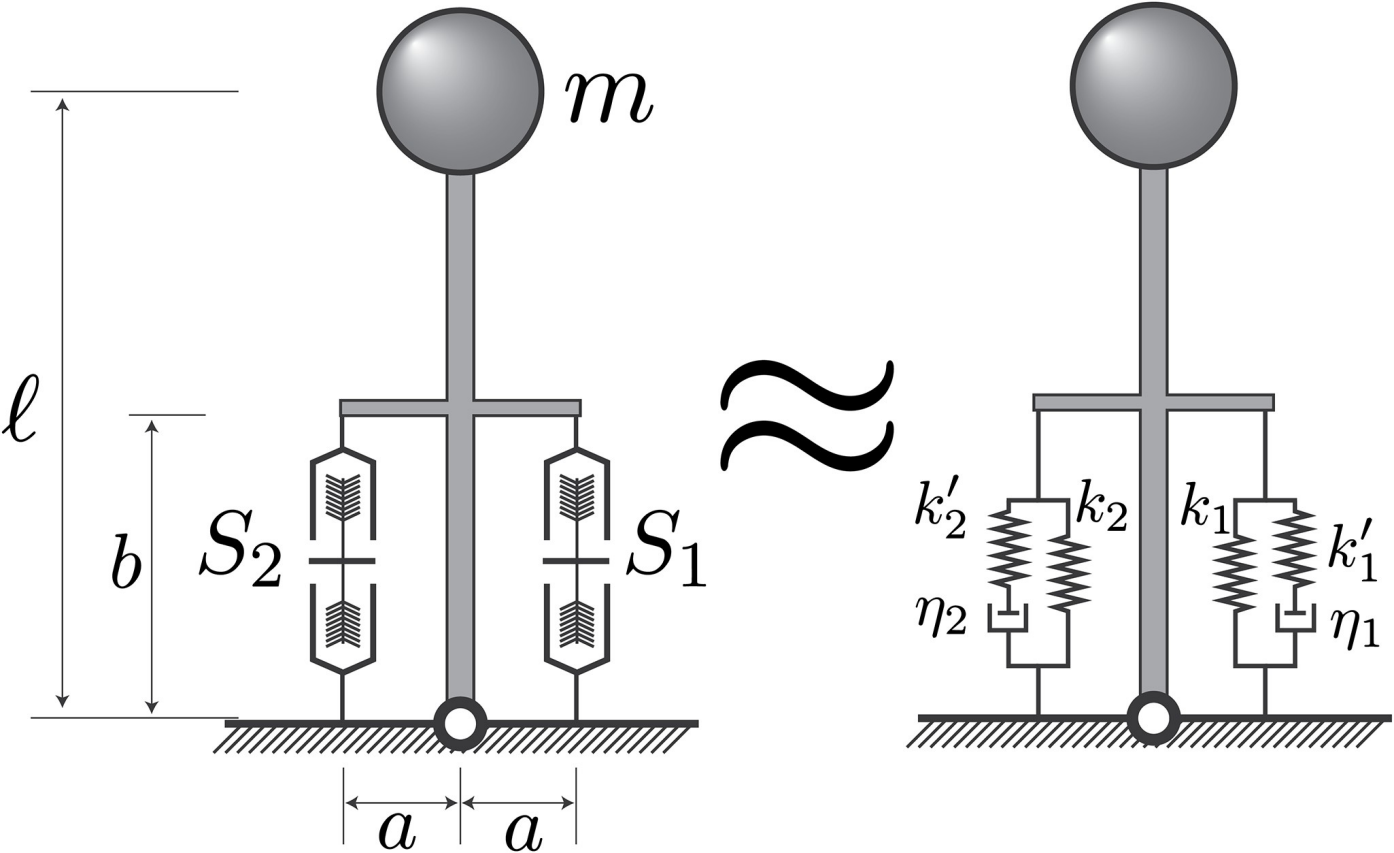
Approximation of the inverted pendulum in this analysis (left) with one supported by standard viscoelastic solid models (right).

We initially presented a stability analysis that classifies equilibria as stable or unstable based on minimizing the potential energy [4,6,15]. Yet our numeric simulations conflicted with the predicted stability. A partial explanation for this mismatch is that our muscle elements do not provide produce conservative forces, which precludes a stability analysis based on potential energy minima. Therefore, we conducted a broader analysis of the inverted pendulum as if it were supported by standard viscoelastic solids, which are a decent approximation to a constant-activation Huxley model (Fig 7). We found that this systems’ stability is independent of the short-range stiffness magnitude and now offer this as an explanation for why the Huxley muscles did not stabilize the inverted pendulum.

We impose that the springs labelled *S*_*i*_ (Fig 7) can be approximated by standard-linear viscoelastic solid elements, whose springs are linear and have the steady-state muscle stiffness (Fig 1) [17,24,25]. On the other hand, the parallel Maxwell elements are linear with spring constant $k_{i}^{\prime }$, and relaxation times $\tau_{i}=\eta_{i}/k_{i}^{\prime }$. This arrangement ensures that if these elements are slowly lengthened, their force-elongation curve will follow the steady-state force-length relation; but, when lengthened quickly, will have a transient short-range stiffness $k_{i}+k_{i}^{\prime }$. Here, *k*_*i*_ is the slope of the tangent to the force-length curve, which we called the static stiffness (c.f. Fig 1). Like before, we let *x*_*i*_ be the total muscle length, and *ξ*_*i*_ be the Maxwell-element springs deflections, so that the muscle tensile forces, *F*_*i*_, can be calculated from the relationships:

$$F_{i}=k_{i}\left(x_{i}-b\right)+f_{L}\left(b\right)+k_{i}^{\prime }\xi_{i}$$ (17a)

$${\dot{\xi }}_{i}={\dot{x}}_{i}-\tau_{i}^{-1}\xi_{i}$$ (17b)

Where *f*_*L*_(*x*) is the steady-state force-length relationship. Next, we apply Newton’s second law to arrive at the equation of motion:

$$ml^{2}\;\ddot{\theta }=-r_{1}F_{1}-r_{2}F_{2}+mgl\;\text{sin}\;\theta$$ (18)

Where *r*_*i*_ are the muscles’ moment arms, and the negatives ensure that positive forces in muscle 1 produces a negative moment. Like before, the muscle lengths, *x*_*i*_, are functions of the joint angle, *θ* (Eqs 2a and 2b). Similarly, the moment arms *r*_*i*_ are the derivatives of *x*_*i*_ with respect to *θ*. At last, we introduce $\omega =\dot{\theta }$ and define the state vector, **q** = (*θ* ω *ξ*_1_ *ξ*_2_)^*T*^. We linearize this system about the upright steady-state, **q**_0_ = (0 0 0 0)^*T*^, which requires that the forces in *S*_1_ and *S*_2_ are equal, yielding the linear system:

$$\frac{\text{d}}{\text{d}t}\left[\begin{array}{c} \theta \\ \omega \\ \xi_{1}\\ \xi_{2} \end{array}\right]=\left[\begin{array}{cccc} 0 & 1 & 0 & 0\\ \frac{\left(mgl-k_{1}a^{2}-k_{2}a^{2}\right)}{ml^{2}} & 0 & -\frac{ak_{1}^{\prime }}{ml^{2}} & \frac{ak_{2}^{\prime }}{ml^{2}}\\ 0 & a & -\tau_{1}^{-1} & 0\\ 0 & -a & 0 & -\tau_{2}^{-1} \end{array}\right]\left[\begin{array}{c} \theta \\ \omega \\ \xi_{1}\\ \xi_{2} \end{array}\right]$$ (19)

Like before, *a* represents the moment arm in the upright position, and *k*_1_ and *k*_2_ are the slopes of the usual force-length expression. We can determine whether the system, of the form $\dot{q}=Aq$, is asymptotically stable by analyzing its eigenvalues, the solutions to the equation 0 = det(λ**I** − **A**). This polynomial is:

$$\begin{array}{ll} 0 & =\lambda^{4}+\left[\tau_{1}^{-1}+\tau_{2}^{-1}\right]\lambda^{3}+\left[\frac{ml^{2}-mgl\tau_{1}\tau_{2}+a^{2}\tau_{1}\tau_{2}\left(\left(k_{1}+k_{1}^{\prime }\right)+\left(k_{2}+k_{2}^{\prime }\right)\right)}{ml^{2}\tau_{1}\tau_{2}}\right]\lambda^{2}\\ & +\left[\frac{a^{2}\tau_{1}\left(\left(k_{1}+k_{1}^{\prime }\right)+k_{2}\right)+a^{2}\tau_{2}\left(\left(k_{2}+k_{2}^{\prime }\right)+k_{1}\right)-mgl\left(\tau_{1}+\tau_{2}\right)}{ml^{2}\tau_{1}\tau_{2}}\right]\lambda \\ & +\left[\frac{a^{2}\left(k_{1}+k_{2}\right)-mgl}{ml^{2}\tau_{1}\tau_{2}}\right] \end{array}$$ (20)

Of course, solving this quartic equation for *λ* would be needlessly laborious, so we employ a useful analytic result. From the Routh-Hurwitz criterion [68], this system will be unstable if any characteristic polynomial coefficients (including the intercept) are negative. Assuming that the short-range stiffnesses ($k_{i}+k_{i}^{\prime }$), time constants (*τ*_*i*_), mass (*m*) and pendulum length (*ℓ*) are all positive, we find that the system is unstable if:

$$k_{1}+k_{2}<\frac{mgl}{a^{2}}$$ (21)

Which is a result similar to the static analysis (Eq 9). We strengthen this claim in Appendix I, where we show that the converse is also true: if *k*_1_ + *k*_2_ > *mgℓ*/*a*^2^, then the Routh-Hurwitz criteria are satisfied, and the pendulum is asymptotically stable. Interestingly, this result does not depend on the short-range stiffness and suggests that the pendulum is unstable if both muscles are on the plateau or descending limbs of the force-length relationship, whereas the ascending limb may be more stable. If this criterion in Eq 21 is met, the pendulum is asymptotically stable, a stronger notion of stability than minimum potential energy suggests, meaning that perturbations converge on the equilibrium rather than oscillate about it.

### Alternative stabilizing mechanisms

Short-range stiffness offers an attractive stabilizing mechanism because it produces a force that resists a perturbation almost instantaneously and without any input from the central nervous system. This effect, along with the amplification of lengthening muscle force from the force-velocity relationship, seems like a plausible stabilizing mechanism for many biomechanical systems, and models that treat short-range stiffness have found that it helps in responding to a perturbation [14,16,30,44]. However, our simulations and analysis are strongly suggestive that short-range stiffness plays a critical supporting role in establishing stability by rapidly dissipating a perturbation, as it cannot stabilize the system on its own. Therefore, another mechanism must be responsible for providing mechanical stability to these systems. In this section, we speculate on a few mechanisms that seem most plausible.

The simplest hypothesis that imposes joint stability might be that stabilizing muscles exist on the ascending limb of the force-length relationship, so they already meet the stability criteria. This property is certainly true for some muscles, in the spine, for example, the multifidus, which Ward et al. [69] found is soundly stationed on the ascending limb in a neutral posture. Similarly, Burkholder and Lieber (2001) [70] found that many human skeletal muscles operate on the ascending limb near the plateau region. Unfortunately, morphometry studies have found that this finding is not always true. The sarcomere lengths in a neutral posture for several muscles, particularly in the cervical spine, are located near the plateau region or on the descending limb [70–73]. These outcomes raise an interesting paradox when combined with our analysis: the active component of muscles on the plateau or descending limbs cannot use force-length properties to stabilize a joint, and yet, many muscles operate in this region and our joints behave in a stable manner. This paradox strongly suggests that the inverted pendulum model may be missing an important feature of muscle mechanics, other viscoelastic soft tissues, joint configuration, posture, or require a controller to maintain stability.

Another hypothesis might be the residual force enhancement or depression phenomena of muscles. In this analysis, we assumed that the steady-state force produced by a lengthened active muscle would, after a transient, tend toward the force-length relationship. However, experimental evidence is abundant at the sarcomere, fibre, and whole muscle levels, which suggests that this is not the case [74–82]. Instead, actively lengthened muscles tend toward more force, called residual force enhancement, and actively shortened muscles tend toward less, called force depression. Interestingly, this change in steady-state force is proportional to the change in stretch, at least on the descending limb [83], and can exceed 50% of the maximum isometric force [81]. This proportionality suggests that its effects may be approximated with another spring, in parallel with the muscles and Maxwell elements, whose resting length is the initial length of the muscle [18,67]. This mechanism would be stabilizing since the proportionality constant would add to the static stiffnesses and, unlike short-range stiffness, not be transient. For our simple inverted pendulum, the stability condition with force enhancement would be:

$$\left(k_{1}+k_{FE}\right)+\left(k_{2}+k_{FE}\right)>\frac{mgl}{a^{2}}$$ (22)

Where *k*_*FE*_ is the proportionality constant relating stretch to force enhancement. This argument demonstrates the plausible stabilizing effect of residual force enhancement and depression but is not comprehensive. Experimentally verifying the potential role of this phenomenon, and its molecular basis, remains an avenue for future work.

Perhaps the most direct hypothesis is that the central nervous system manages joint stability through motor control and reflexes [43,84,85]. Using the spine as an example, the small muscles of the spine, like the rotatores and intertransversarii, have some of the densest clusters of muscle spindles in the human body, allowing them to quickly transmit postural signals to the spinal cord [86,87]. Further, some computer studies have obtained stable cervical spine behaviour using controls that respond to vestibular reflexes with Hill-type muscle models that typically omit short-range stiffness [88]. The most compelling evidence for this hypothesis, at least for the spine, comes from Moorhouse and Granata (2007) [43], who used a system identification technique with experimental data to determine that spinal reflexes accounted for 42% of the total stabilizing trunk stiffness. While short-range stiffness may still play a role in this paradigm, it may function as a damper that constrains and dissipates the perturbation and buys the central nervous system time to respond to a disturbance.

## Limitations

There are still some modelling assumptions in this work that may diminish its generalizability. The first is the rigid-tendon assumption that was made throughout this analysis. Since tendons are in series with the contractile element, the compliance of the overall musculotendon unit is the sum of tendon and contractile element compliances. This means that the stiffness of the overall unit is limited by the least-stiff element in series. At low muscle forces this will be the contractile element; but at higher forces the overall stiffness is limited by the tendon. Therefore, the rigid tendon approximation used here has produced a supraphysiological stiffness, the best-case scenario for such a phenomenon to stabilize the inverted pendulum model.

We can demonstrate the stiffness limiting effect of the tendon as follows. For forces in the linear-region of the tendon’s force-length curve, the tendon has linear stiffness *k*_*T*_. Then, using Eq 10 for that of the contractile element, the stiffness of the serially arranged musculotendon unit is:

$$k_{MT}=\frac{k_{T}F}{F+\left(\frac{k_{T}x_{0}}{q}\right)}$$ (23)

In other words, the overall stiffness starts off being well-approximated by Eq 10, but asymptotically approaches the tendon’s stiffness as the muscle force increases. When the muscle force is equal to $\frac{k_{T}x_{0}}{q}$, the overall stiffness will be half that of the tendon. This diminishing stiffness returns as force increases agrees with previous experimental [30,89] and theoretical work [90,91], and is suggestive that an infinitely rigid tendon provides the best-case scenario for contractile element stiffness to contribute to stability.

The second consideration was the decision to omit the force-length curve in the distribution moment implementation of the muscle, including the passive properties. There is no question that passive properties have a substantial effect on the force predicted in musculoskeletal models [92], and ignoring it during numeric simulations may have omitted an important detail. Despite this decision, the role of these two properties was clarified by the analytical analysis. That analysis revealed that a positive slope on the combined force-length curve, active or passive, contributes to the stability of the inverted pendulum.

A potential argument regarding the timescale of the pendulum’s response can be made. The short-range stiffness effect clearly dampens the initial perturbation, limits the initial angular excursion, and reduces the angular velocity. This is particularly significant at high activations, where the pendulum takes tens of seconds to finally topple over—a trivial timespan for the central nervous system to intervene and restore the pendulum to its upright position. However, this practical notion of stability contrasts with the mathematical definition used throughout this manuscript. The fact that the pendulum is ’practically’ stable for a given set of muscle activations warrants further investigation.

Finally, equipped with the system dynamics, as well as the differential equations governing the DM approximations, it might be tempting to conduct a stability analysis directly on these equations. We chose not to for two reasons. The first is that the DM approximation equations are not readily differentiable, rendering them unsuitable for analytic stability analysis. As an alternative, a numerical stability analysis would have been required, which leads to our second rationale. The resulting numerical analysis would lack the mechanical intuition obtained by first reducing the Huxley model into a rheological model. We believe this analysis substantiates the otherwise ‘ad-hoc’ placement of springs and dashpots in the Hill model to endow it with short-range stiffness properties.

## Conclusions

Our simulations suggested that muscle short-range stiffness cannot be solely responsible for joint stability, even for modest perturbations. However, with subsequent analysis of the Huxley model, we argue that the transient-nature of the rise in force from short-range stiffness is responsible for insufficient stability. Ultimately, short-range stiffness of active muscle does not behave like a typical spring, as previously assumed; but rather, like a Maxwell element. The damping that results from short-range stiffness may slow down the mechanical response and allow the central nervous system time to react and stabilize the joint. Other mechanisms, such as residual force enhancement or spinal reflexes, may play a more substantial role in joint stability. Overall, joint stability is due to a combination of factors and cannot be reduced to simply a problem of muscle stiffness.

## Supporting information

S1 File A document containing a derivation of short-range stiffness using the Huxley model, considering an arrangement of springs in series and parallel, and approximating the transient behaviour as a Maxwell element. (PDF)

S2 File A zip-folder containing animations of each of the perturbations for the various conditions tested in this manuscript. (ZIP)

S1 Appendix (DOCX)
