# Supplementary material for: Muscle short-range stiffness behaves like a maxwell element, not a spring: Implications for joint stability
Source: PLoS One. 2024 Aug 14;19(8):e0307977. doi: 10.1371/journal.pone.0307977 (PMC11324116; doi:10.1371/journal.pone.0307977)
Supplement: S1 File — (PDF) [file pone.0307977.s001.pdf]

Estimating the Huxley Model Step Response with Linear Viscoelastic Theory

Jeff Barrett

October 2023

1 Introduction

Previous experiments have noted that the response of activated muscle samples to a rapid stretch behaves, at least phenomenologically, similarly to a Maxwell element. Theoretically, this response is well-characterized by Huxley's muscle model. However, to the authors' knowledge, there has yet to be an analysis exploring the connection between these two descriptions. Therefore, this supplemental material demonstrates how the Maxwell-model behaviour can be derived from the Huxley model.

2 Background

The Huxley model under analysis here is the first-order partial differential equation, which governs the time-evolution of cross-bridges in a half-sarcomere:

$$\frac{\partial n}{\partial t} - v\frac{\partial n}{\partial x} = F(s)f(x) - \left(f(x) + g(x)\right)n(x,t) \tag{1}$$

Where $n(x,t)$ represents the density of bound myosin cross-bridges strained by x, so that $n(x,t)\Delta x$ can be thought of as, roughly, the proportion of cross-bridges lengthened between x and $x + \Delta x$ of their resting position.

The functions $f(x)$ and $g(x)$ describe the binding and unbinding rates, respectively. They are functions of displacement, x, to represent their dependence on myosin strain. Canonically, they are taken as piecewise linear functions:

$$f(x) = \begin{cases} f_1 x & x \in (0,h) \\ 0 & \text{otherwise} \end{cases} \qquad g(x) = \begin{cases} g_0 & x \in (-\infty, 0) \\ g_1 x & x \in [0,h) \\ g_2(x-h) + h(g_1 - g_2) & x \in [h, \infty) \end{cases} \tag{2}$$

Here h is the "*characteristic bond length*" for myosin. Finally, we let s be the length of the sarcomere, and $F(s)$ is the force-length curve, famously determined by Gordon and Huxley (1966).

2.1 Short Range Stiffness

An appealing aspect of the Huxley model is that it inherently predicts the "short-range stiffness" phenomenon. First, consider the steady-state isometric cross-bridge distribution is given by:

$$n_{ss}(x) = \frac{f(x)}{f(x)+g(x)} = \begin{cases} \varphi & x \in (0,h) \\ 0 & \text{otherwise} \end{cases} \tag{3}$$

Where we have defined $\varphi = \frac{f_1}{f_1+g_1}$. Similarly the force is:

$$P_0 = \Gamma \int_{-\infty}^{\infty} x n_{ss}(x)\, \mathrm{d}x = \frac{1}{2}\Gamma\varphi h^2 \tag{4}$$

Now we consider how this force changes under a small length increase of the muscle belly. Let X be the length of the muscle belly, with X_0 being that in a reference position, and N_s be the number of sarcomeres in series. In the reference position, the sarcomeres are of length s_0, given by:

$$s_0 = \frac{X_0}{N_s}$$

Then the sarcomere length when the muscle belly has length X is, from the conservation of sarcomeres:

$$\frac{s_0}{X_0} = \frac{s}{X}$$

These relationships ensure that if X is lengthened by ΔX, then the sarcomeres experience a lengthening by:

$$\Delta s = \frac{s_0}{X_0}\Delta X$$

And so the half-sarcomeres experience a displacement of:

$$\Delta x = \frac{1}{2}\Delta s = \frac{s_0}{2X_0}\Delta X \tag{5}$$

The net effect is that if the muscle is lengthened by ΔX, then the distribution shifts by Δx. Thus, we expect to see a change in force given by:

$$P = \Gamma \int_{-\infty}^{\infty} x n_{ss}(x-\Delta x)\, \mathrm{d}x \tag{6}$$

For small Δx, we can expand this to first-order:

$$P = \Gamma \int_{-\infty}^{\infty} \left(x n_{ss}(x) - x\frac{\partial n_{ss}}{\partial x}\Delta x \right) \mathrm{d}x$$

Expanding this out and using integration by parts on the second term we simply get:

$$P = P_0 + \Gamma \Delta x \int_{-\infty}^{\infty} n_{ss}(x)\, \mathrm{d}x$$

The integral can be easily evaluated to φh. Simplifying this gives:

$$\Delta P = P - P_0 = \Gamma\varphi h \Delta x$$

Now we substitute Δx for ΔX:

$$\Delta P = \Gamma \varphi h \frac{s_0}{2X_0} \Delta X$$

And from here we define the *short-range stiffness*, denoted K_0, of the muscle belly to be:

$$K_0 = \frac{\Delta P}{\Delta X} = \Gamma \varphi h \frac{s_0}{2X_0} \tag{7}$$

The last step to relate this to force is to recognize that we can use Equation 4 to relate this short-range stiffness to force:

$$K_0 = \frac{s_0}{h} \frac{P_0}{X_0} = q \frac{P_0}{X_0} \tag{8}$$

Which is the expression we have in the manuscript.

2.1.1 A More Intuitive Description

This theoretical q deviates from what has been widely used in the literature for the stiffness of the contractile element, typically by an order of magnitude. Interestingly, we can derive the same q instead, considering a very large population of myosin cross-bridges without appealing to the form of Huxley's equations.

Consider a single myofibril consisting of N identical sarcomeres arranged in series. Each half-sarcomere contains a discrete population of M cross-bridges bound to actin; they act as M identical springs with constant k_x arranged in parallel, each with displacements $x_1, x_2, \ldots, x_M$. Importantly, their average displacement is $\mu = \frac{1}{M}\sum_{i=1}^{M} x_i$. Despite the simplicity of this arrangement, we can make the following observations:

(a) The total force in the half-sarcomere (P_0) equals the half-sarcomere's stiffness (k_s) times the average stretch in the cross-bridge (μ). To see why this is true, recall that the half-sarcomere stiffness is $M \times k_x$ because each cross-bridge acts in parallel. The total force is the sum of forces in each of the springs, which gives:

$$P = \sum_{i=1}^{M} k_x x_i = k_x \sum_{i=1}^{M} x_i = M k_x \left(\frac{1}{M} \sum_{i=1}^{M} x_i \right) = k_s \mu$$

(b) The stiffness of the whole myofibril (K) is proportional to the force (P_0) divided by the number of sarcomeres in series (N). If the myofibril consists of N serially arranged sarcomeres, then it consists of $2N$ serially arranged half-sarcomeres. From **(a)**, we have that the stiffness of the half-sarcomeres is $k_s \mu$. Serially arranged elements give a stiffness of:

$$\frac{1}{K} = \sum_{i=1}^{2N} \frac{1}{k_s} = \sum_{i=1}^{2N} \frac{\mu}{P_0} = 2N \frac{\mu}{P_0}$$

And so,

$$K = \frac{1}{2\mu N} P_0$$

(c) The number of sarcomeres in series is equal to the myofibrillar length X_0 divided by the length of the sarcomeres at that length, s_0. This gives:

$$K = \frac{s_0}{2\mu X_0} P_0$$

(d) Steps **(a)** to **(c)** are generally valid and have made no assumptions about the way the myosin displacements are distributed. This distribution will affect μ, the average deflection. If they are uniformly distributed between 0 and h, then the average is $\mu = h/2$. Plugging this in gives the expression:

$$K = \frac{s_0}{hX_0} P_0 = \frac{s_0}{h} \frac{P_0}{X_0} = q \frac{P_0}{X_0}$$

Which is the same expression derived from Huxley's equations.

3 Capturing the Transient Behaviour

Recognizing the short-range stiffness effect was instrumental in uncovering the sliding filament mechanism of muscular contraction. However, the above analysis only considered what happens immediately after lengthening and not the time-course of force over time after the muscle has been lengthened. Thus, in this section, we derive an expression showing that the transient behaviour can be roughly approximated as a Maxwell element.

3.1 The Very Simplified Case

We begin with a very simplified case, noting that the full-model derivation will come a bit later. First, it's necessary to understand how the Huxley model results in behaviour akin to a Maxwell element. For this, we omit the force-length curve, $F(s) = 1$ for all s, and we further simplify the binding and unbinding rates so that the unbinding rates for large x is constant. We justify the latter by suggesting that we only lengthen the sarcomere by a tiny amount, Δs, so we will not be bothered tremendously by the behaviour of the binding coefficients for values of x larger than h. Taken together, our approximate binding and unbinding rates are now:

$$\hat{f}(x) = \begin{cases} \varphi \lambda x & x \in (0, 1) \\ 0 & \text{otherwise} \end{cases} \qquad \hat{g}(x) = \begin{cases} \lambda_0 & x \in (-\infty, 0) \\ \lambda(1-\varphi)x & x \in [0, 1) \\ \lambda & x \in [1, \infty) \end{cases} \tag{9}$$

With these binding rates, the steady-state solution is given by:

$$\tilde{n}(x) = \frac{\hat{f}(x)}{\hat{f}(x) + \hat{g}(x)} = \begin{cases} \varphi & 0 \le x \le 1 \\ 0 & \text{otherwise} \end{cases} \tag{10}$$

We can further introduce the function $\tau(x) = (\hat{f}(x) + \hat{g}(x))^{-1}$. We can use this solution to rewrite Huxley's Equation as:

$$\frac{\partial n}{\partial t} - v \frac{\partial n}{\partial x} = \frac{1}{\tau(x)} \left(\tilde{n}(x) - n(x, t) \right)$$

Suppose that the half-sarcomere is now lengthened by Δx. We now have the initial value problem:

$$\frac{\mathrm{d}n}{\mathrm{d}t} = \frac{1}{\tau(x)} \left(\tilde{n}(x) - n(x, t) \right) \tag{11}$$

$$n(x, 0) = \tilde{n}(x - \Delta x) \tag{12}$$

This is a series of differential equations indexed by x. However, we know that $n(x, t)$ will remain zero for $x < 0$ and $x > 1 + \Delta x$. Further, it is still in steady-state and equal to φ over the range $\Delta x < x < 1$. That means we only need to solve it over two possible ranges: $0 < x < \Delta x$ and $1 < x < 1 + \Delta x$.

The solution for lengthening is:

$$n_L(x,t) = \begin{cases} \varphi(1-e^{-\lambda t}) & x \in (0, \Delta x) \\ \varphi & x \in (\Delta x, 1) \\ \varphi e^{-\lambda t} & x \in (1, 1+\Delta x) \\ 0 & \text{otherwise} \end{cases} \tag{13}$$

We now evaluate the force-over-time resulting from this rapid lengthening experiment. Noting that this is a bit of a slog because we need to evaluate three terms in the integral:

$$P(t) = \Gamma \int_{-\infty}^{\infty} x n_L(x,t)\,\mathrm{d}x \tag{14}$$

$$= \Gamma \int_{0}^{\Delta x} x n_L(x,t)\,\mathrm{d}x + \Gamma \int_{\Delta x}^{1} x n_L(x,t)\,\mathrm{d}x + \Gamma \int_{1}^{1+\Delta x} x n_L(x,t)\,\mathrm{d}x \tag{15}$$

Plugging in the solution we found and simplifying everything, we simply find that:

$$P(t) = \left(\Gamma \varphi e^{-\lambda t}\right) \Delta x + P_0 \tag{16}$$

Where $P_0 = \varphi/2$ is the force before the lengthening began. Initially, upon lengthening, the force rises, but then exponentially decays to a constant value. The first term, which does the relaxation, resembles the relaxation function for a Maxwell element. If is for this reason that we suggest that short-range stiffness behaves like a Maxwell element. It is, in fact, equivalent to a Maxwell element arranged in parallel with a constant-force spring.

4 Approximating Short-Range Stiffness with Linear Viscoelasticity

Here, we analyze the response to a rapid length change experiment and consider the overall response of the Huxley model. We make some variable substitutions because we wish to generalize up to the whole muscle level. We let $\xi = x/h$, and $u = v/h$.

$$\frac{\partial n}{\partial t} - u\frac{\partial n}{\partial \xi} = F(X)f(\xi) - \left(f(\xi) + g(\xi)\right) n(\xi, t)$$

Like before, the functions $f(\xi)$ and $g(\xi)$ describe the binding and unbinding rates, respectively. They are functions of displacement, ξ, to represent their dependence on myosin strain. Canonically, they are taken as piecewise linear functions:

$$f(x) = \begin{cases} f_1\xi & \xi \in (0,1) \\ 0 & \text{otherwise} \end{cases} \qquad g(x) = \begin{cases} g_0 & \xi \in (-\infty, 0) \\ g_1\xi & \xi \in [0,1) \\ g_2(\xi-1) + (g_1 - g_2) & \xi \in [1, \infty) \end{cases}$$

Furthermore, $F(X)$ is the force-length relationship, and X is the length of the full contractile tissue, with X_0 being that length in a reference state. This has a steady-state solution:

$$N(\xi, X) = \frac{F(X)f(\xi)}{f(\xi)+g(\xi)} = \begin{cases} \frac{F(X)f_1}{f_1+g_1} & 0 \le \xi \le 1 \\ 0 & \text{otherwise} \end{cases}, \qquad \sigma_{\text{iso}}(X) = \Gamma \int_{-\infty}^{\infty} \xi N(\xi, X)\,\mathrm{d}\xi = \Gamma \frac{F(X)f_1}{2(f_1+g_1)}$$

Which we can use to rewrite the original equation as:

$$\frac{\partial n}{\partial t} - u\frac{\partial n}{\partial \xi} = \frac{1}{\tau(\xi)}\left(N(\xi, X) - n(\xi, t)\right)$$

Where we have defined $\tau(\xi) = (f(\xi) + g(\xi))^{-1}$. Now we perturb the position by ΔX. The half-sarcomere, will experience a change of $\Delta x = \frac{\Delta X}{2X} s$, which shifts out distribution by $\Delta\xi = \frac{\Delta x}{h}$ so we end up solving the IVP:

$$\frac{\partial n}{\partial t} = \frac{1}{\tau(\xi)}\left(N(\xi, X + \Delta X) - n(\xi, t)\right)$$
$$n(\xi, 0) = N(\xi - \Delta\xi, X)$$

This is essentially just an ordinary differential equation indexed by x. The solution is:

$$n(\xi, t) = \left(N(\xi - \Delta\xi, X) - N(\xi, X + \Delta X)\right)\exp\left(-\frac{t}{\tau(\xi)}\right) + N(\xi, X + \Delta X)$$

Linear approximations:

$$n(\xi, t) = -\left(\Delta\xi\frac{\partial N}{\partial \xi} + \Delta X\frac{\partial N}{\partial X}\right)\exp\left(-\frac{t}{\tau(\xi)}\right) + N(\xi, X) + \Delta X\frac{\partial N}{\partial X}$$

Then:

$$n(\xi, t) = -\left(\Delta\xi\frac{\partial N}{\partial \xi} + \Delta X\frac{\partial N}{\partial F}F'(X)\right)\exp\left(-\frac{t}{\tau(\xi)}\right) + N(\xi, X) + \Delta X\frac{\partial N}{\partial F}F'(X)$$

Now we do term by term. For brevity, let $\tau_1 = \tau(1) = (f_1 + g_1)^{-1}$, and $F'(X) = k$, the slope of the force-length curve.

$$\begin{aligned}
\text{first} &= \Gamma\int_{-\infty}^{\infty} \xi\Delta\xi\frac{\partial N}{\partial \xi}e^{-t/\tau(\xi)}\,\mathrm{d}\xi \\
&= \Gamma F(X)\frac{f_1}{2(f_1 + g_1)}\Delta\xi\int_{-\infty}^{\infty} \xi\left(\delta(\xi) - \delta(\xi - 1)\right)e^{-t/\tau(\xi)}\,\mathrm{d}\xi \\
&= -\frac{\Gamma f_1 F(X)}{2(f_1 + g_1)}\Delta\xi e^{-t/\tau_1}
\end{aligned}$$

Where we have added the factor of 1/2 to account for the deltas being at the boundaries of the Heaviside step functions in the steady-state solution. And:

$$\begin{aligned}
\text{second} &= \Gamma\int_{-\infty}^{\infty} \xi k\Delta X\frac{\partial N}{\partial F}e^{-t/\tau(\xi)}\,\mathrm{d}\xi \\
&= \frac{k\Gamma f_1\Delta X}{f_1 + g_1}\int_{0}^{1} \xi\exp\left(-\frac{t}{\tau_1\xi}\right)\mathrm{d}\xi \\
&= \frac{k\Gamma f_1\Delta X}{2(f_1 + g_1)}\left(\left(1 - \frac{t}{\tau_1}\right)e^{-t/\tau_1} - \frac{t^2}{\tau_1^2}\mathrm{Ei}\left(-\frac{t}{\tau_1}\right)\right)
\end{aligned}$$

Then:

$$\text{third term} = \Gamma\int_{-\infty}^{\infty} \xi N(\xi, X)\,\mathrm{d}\xi = \sigma(t)$$

And, finally:

$$\begin{aligned}
\text{fourth} &= k\Gamma\Delta X \int_{-\infty}^{\infty} \xi \frac{\partial N}{\partial F}\,\mathrm{d}\xi \\
&= \frac{k\Gamma f_1 \Delta X}{f_1+g_1}\int_0^1 \xi\,\mathrm{d}\xi \\
&= \frac{k\Gamma f_1 \Delta X}{2(f_1+g_1)}
\end{aligned}$$

Combining all these together, we have:

$$\sigma(t+\Delta t)-\sigma(t) = \frac{\Gamma f_1 F(X)}{2(f_1+g_1)}\Delta\xi e^{-t/\tau_1} - \frac{k\Gamma f_1\Delta X}{2(f_1+g_1)}\left(\left(1-\frac{t}{\tau_1}\right)e^{-t/\tau_1} - \frac{t^2}{\tau_1^2}\mathrm{Ei}\left(-\frac{t}{\tau_1}\right)\right) + \frac{k\Gamma f_1 \Delta X}{2(f_1+g_1)}$$

Using that $\Delta x = s_0\Delta X/(2hX_0)$ this gives:

$$\begin{aligned}
\Delta\sigma(t) &= \left[\frac{\Gamma f_1 s_0 F(X)}{4h(f_1+g_1)X_0}e^{-t/\tau_1} - \frac{k\Gamma f_1}{2(f_1+g_1)}\left(\left(1-\frac{t}{\tau_1}\right)e^{-t/\tau_1} - \frac{t^2}{\tau_1^2}\mathrm{Ei}\left(-\frac{t}{\tau_1}\right)\right) + \frac{k\Gamma f_1}{2(f_1+g_1)}\right]\Delta X \\
&= \frac{\Gamma f_1}{2(f_1+g_1)}\left[\frac{s_0F(X)}{2hX_0}e^{-t/\tau_1} - k\left(\left(1-\frac{t}{\tau_1}\right)e^{-t/\tau_1} - \frac{t^2}{\tau_1^2}\mathrm{Ei}\left(-\frac{t}{\tau_1}\right)\right) + k\right]\Delta X \\
&= \frac{\Gamma f_1}{2(f_1+g_1)}\left[\frac{s_0F(X)}{2hX_0}e^{-t/\tau_1} + F'(X)\left(1+\frac{t^2}{\tau_1^2}\mathrm{Ei}\left(-\frac{t}{\tau_1}\right) + \left(\frac{t}{\tau_1}-1\right)e^{-t/\tau_1}\right)\right]\Delta X
\end{aligned}$$

Now we apply the principle of superposition from Linear Viscoelastic Theory, this ends gives:

$$\sigma(t) = \int_0^t E(t-s)\frac{\mathrm{d}X}{\mathrm{d}t}\,\mathrm{d}s$$

Where the relaxation function is:

$$E(t) = \sigma_{\max}\left[\frac{s_0F(X)}{2hX_0}e^{-t/\tau_1} + F'(X)\left(1+\frac{t^2}{\tau_1^2}\mathrm{Ei}\left(-\frac{t}{\tau_1}\right) + \left(\frac{t}{\tau_1}-1\right)e^{-t/\tau_1}\right)\right] \tag{17}$$

We can approximate:

$$\frac{t^2}{\tau_1^2}\mathrm{Ei}\left(-\frac{t}{\tau_1}\right) \approx -\frac{t}{\tau_1}e^{-t/\tau_1}$$

Which gives the simplified relaxation function:

$$E(t) = \sigma_{\max}\left[\left(\frac{s_0F(X)}{2hX_0} - F'(X)\right)e^{-t/\tau_1} + F'(X)\right]$$

If we further assume that $\frac{s_0F(X)}{2hX_0} \gg F'(X)$, then we can further simplify this to:

$$E(t) = \left(\frac{s_0\sigma_{\max}F(X)}{2hX_0}\right)e^{-t/\tau_1} + \sigma_{\max}F'(X)$$

Finally, we introduce $k = \sigma_{\max}F'(X)$, and $k' = \frac{s_0\sigma_{\max}F(X)}{2hX_0}$, to arrive at:

$$E(t) = k'e^{-t/\tau_1} + k \tag{18}$$

And this is precisely the relaxation function for a standard viscoelastic solid model. This also has the correct form of the short-range stiffness used in the manuscript.
