# Supplementary material for: Muscle short-range stiffness behaves like a maxwell element, not a spring: Implications for joint stability
Source: PLoS One. 2024 Aug 14;19(8):e0307977. doi: 10.1371/journal.pone.0307977 (PMC11324116; doi:10.1371/journal.pone.0307977)
Supplement: S1 Appendix — (DOCX) [file pone.0307977.s003.docx]

**Appendix I: Necessary and Sufficient Condition for Stability**

It is helpful to first introduce some new parameters in order to make following algebra simpler. Let $I=m\mathcal{l}^{2}, \kappa_{1}=\frac{k_{1}^{'}r^{2}}{I}$, and $\kappa_{2}=\frac{k_{2}^{'}r^{2}}{I}$. Note that in assuming that the mass, length, short-range stiffnesses and relaxation times are all positive implies that $I>0, \kappa_{i}>0$ and $\tau_{i}>0$. Finally, we let $\varepsilon=(r^{2}\left( k_{1}+k_{2} \right)-mg\mathcal{l})/I$ and note that $\varepsilon>0$ is equivalent to our proposed stability condition, $k_{1}+k_{2}>mg\mathcal{l}/r^{2}$. With these substitutions, the polynomial becomes:

$$0=\lambda^{4}+\left( \tau_{1}^{-1}+\tau_{2}^{-1} \right)\lambda^{3}+\left( \kappa_{1}+\kappa_{2}+\varepsilon+\frac{1}{\tau_{1}\tau_{2}} \right)\lambda^{2}+\left( \frac{\kappa_{1}+\varepsilon}{\tau_{2}}+\frac{\kappa_{2}+\varepsilon}{\tau_{1}} \right)\lambda+\frac{\varepsilon}{\tau_{1}\tau_{2}}$$

For brevity, we rename the coefficients for reference in the proof:

$$0=\lambda^{4}+a_{3}\lambda^{3}+a_{2}\lambda^{2}+a_{1}\lambda+a_{0}$$

**Proposition A.1**: This system is stable if and only if $\varepsilon>0$.

*Proof:* First we go in the reverse direction and assume the system is stable. Then, by the Routh-Hurwitz criterion, all the coefficients of Equation A-1 are positive. Since we have $a_{0}=\frac{\varepsilon}{\tau_{1}\tau_{2}}>0$, this implies that $\varepsilon>0$.

Now in the reverse direction, we assume that $\varepsilon>0$. Then, since the other parameters are all positive, we have that all the coefficients of the polynomial will be positive as they are the sum of positive real values. The final condition for the Routh-Hurwitz criterion is that:

$$\Delta=a_{3}a_{2}a_{1}-\left( a_{1}^{2}+a_{0}a_{3}^{2} \right)>0$$

Evaluating this with our parameters yields:

$$\Delta=\frac{\kappa_{1}^{2}}{\tau_{1}\tau_{2}}+\frac{\kappa_{1}\kappa_{2}}{\tau_{2}^{2}}+\frac{\kappa_{1}\kappa_{2}}{\tau_{1}^{2}}+\frac{\kappa_{1}}{\tau_{1}\tau_{2}^{3}}+\frac{\kappa_{1}}{\tau_{1}^{2}\tau_{2}^{2}}+\frac{\kappa_{2}^{2}}{\tau_{1}\tau_{2}}+\frac{\kappa_{2}}{\tau_{1}^{2}\tau_{2}^{2}}+\frac{\kappa_{2}}{\tau_{1}^{3}\tau_{2}}+\left( \frac{\kappa_{2}}{\tau_{2}^{2}}+\frac{\kappa_{1}+\kappa_{2}}{\tau_{1}\tau_{2}}+\frac{\kappa_{1}}{\tau_{1}^{2}} \right)\varepsilon$$

Which, although complicated, is explicitly the sum of positive real numbers and is therefore positive. Therefore, if $\varepsilon>0$ the Routh-Hurwitz criteria are satisfied and the system is stable. Since we have this in both directions, we conclude that $\varepsilon>0$ is a necessary and sufficient condition for stability.
